# Assessing the Role of Ependymal and Vascular Cells as Sources of Extracellular Cues Regulating the Mouse Ventricular-Subventricular Zone Neurogenic Niche

**DOI:** 10.3389/fcell.2022.845567

**Published:** 2022-04-05

**Authors:** Sabrina Quaresima, Arif Istiaq, Hirofumi Jono, Emanuele Cacci, Kunimasa Ohta, Giuseppe Lupo

**Affiliations:** ^1^ Department of Biology and Biotechnology “C. Darwin”, Sapienza University of Rome, Rome, Italy; ^2^ Department of Stem Cell Biology, Faculty of Arts and Science, Kyushu University, Fukuoka, Japan; ^3^ Department of Brain Morphogenesis, Institute of Molecular Embryology and Genetics, Graduate School of Medical Sciences, Kumamoto University, Kumamoto, Japan; ^4^ Department of Pharmacy, Kumamoto University Hospital, Kumamoto, Japan; ^5^ Department of Clinical Pharmaceutical Sciences, Graduate School of Pharmaceutical Sciences, Kumamoto University, Kumamoto, Japan

**Keywords:** neurogenesis, neurogenic niche, neural stem/progenitor cells, ependymal cells, endothelial cells, pericytes, cell signaling

## Abstract

Neurogenesis persists in selected regions of the adult mouse brain; among them, the ventricular-subventricular zone (V-SVZ) of the lateral ventricles represents a major experimental paradigm due to its conspicuous neurogenic output. Postnatal V-SVZ neurogenesis is maintained by a resident population of neural stem cells (NSCs). Although V-SVZ NSCs are largely quiescent, they can be activated to enter the cell cycle, self-renew and generate progeny that gives rise to olfactory bulb interneurons. These adult-born neurons integrate into existing circuits to modify cognitive functions in response to external stimuli, but cells shed by V-SVZ NSCs can also reach injured brain regions, suggesting a latent regenerative potential. The V-SVZ is endowed with a specialized microenvironment, which is essential to maintain the proliferative and neurogenic potential of NSCs, and to preserve the NSC pool from exhaustion by finely tuning their quiescent and active states. Intercellular communication is paramount to the stem cell niche properties of the V-SVZ, and several extracellular signals acting in the niche milieu have been identified. An important part of these signals comes from non-neural cell types, such as local vascular cells, ependymal and glial cells. Understanding the crosstalk between NSCs and other niche components may aid therapeutic approaches for neuropathological conditions, since neurodevelopmental disorders, age-related cognitive decline and neurodegenerative diseases have been associated with dysfunctional neurogenic niches. Here, we review recent advances in the study of the complex interactions between V-SVZ NSCs and their cellular niche. We focus on the extracellular cues produced by ependymal and vascular cells that regulate NSC behavior in the mouse postnatal V-SVZ, and discuss the potential implication of these molecular signals in pathological conditions.

## Introduction

Neurogenesis is the process leading to the formation of new neurons through the proliferation of neural stem/progenitor cells (NSPCs) and the subsequent neuronal differentiation of their progeny. In mice, most of the neurons present in the adult central nervous system (CNS) are produced during embryogenesis by radial glial cells (RGCs), a highly proliferative neural stem cell (NSC) population found throughout the embryonic neural tube (Anthony et al., 2004; Kriegstein and Alvarez-Buylla, 2009). Embryonic neurogenesis peaks during the second or the third week of gestation, depending on the specific CNS region, followed by the progressive depletion of the RGC pool during late embryogenesis and early postnatal life, due to their terminal differentiation into ependymal cells or glial cells (Spassky et al., 2005; Kessaris et al., 2008). In the embryonic murine telencephalon, during the neurogenic peak, a RGC subpopulation produces daughter cells that exit the cell cycle and give rise to ependymal cells or to B1 cells, a subtype of astroglial cells that retains NSC properties (Fuentealba et al., 2015; Furutachi et al., 2015; Ortiz-Álvarez et al., 2019; Redmond et al., 2019). The former cell population never re-enters the cell cycle in physiological conditions and forms the ependymal ventricular zone lining the lateral ventricle (LV). Although B1 cells are located in the subventricular zone underneath the ependymal layer, their apical processes extend in the ventricular zone; they remain largely quiescent until birth, but maintain a latent potential for self-renewal and neuronal production under appropriate stimuli (Doetsch et al., 1999; Obernier and Alvarez-Buylla, 2019). Thus, the ventricular-subventricular zone (V-SVZ) underlying the LV acts as a neurogenic niche harboring a long-lasting NSC population in the postnatal and in the adult mouse brain.

B1 cells can exist in a quiescent, non-proliferative state (qNSCs), in which they are generally thought to be arrested in G0; however, postnatally, they can also undergo activation (aNSCs), entering the cell cycle to self-renew and generate progeny that progresses through the neurogenic lineage to give rise to new neurons (Codega et al., 2014; Obernier and Alvarez-Buylla, 2019). The divisions of aNSCs appear to be mostly symmetric, leading to either self-renewal, by forming two B1 cells, or to the production of two transient amplifying progenitors (TAPs; also known as C cells) (Obernier et al., 2018). TAPs usually divide 3–4 times, giving rise to neuroblasts (NBs, also known as A cells), which exit the cell cycle, migrate to the olfactory bulb and differentiate into interneurons that integrate into olfactory-related neuronal circuitry (Lim and Alvarez-Buylla, 2016). It has been estimated that 1 in 5 aNSC divisions lead to self-renewal, the rest being TAP-forming divisions that consume the NSC pool, potentially causing the marked decrease in B1 cell number that has been observed at early postnatal stages (Obernier et al., 2018). The pool of proliferating NSPCs (aNSCs, TAPs and proliferative NBs) and the neuronal output of the V-SVZ also show a remarkable age-dependent reduction, which happens sharply during the first 6–12 months of postnatal life, followed by a shallower decrease at older ages (Enwere et al., 2004; Luo et al., 2006; Apostolopoulou et al., 2017; Lupo et al., 2019; Navarro Negredo et al., 2020). Moreover, aging is associated with an increase of NSC quiescence and a decrease of NSC activation, which may help to prevent the exhaustion of the adult qNSC population (Bast et al., 2018; Kalamakis et al., 2019; Xie et al., 2020). Even in the aged V-SVZ, it is possible to find a reservoir of qNSCs, which may be capable of activation *in vivo* or *in vitro*, albeit with reduced efficiency (Ahlenius et al., 2009; Kalamakis et al., 2019; Xie et al., 2020). These observations suggest that both NSC depletion and increased NSC quiescence contribute to the age-related neurogenic decline of the postnatal V-SVZ.

Besides NSCs and their neurogenic progeny, several non-neurogenic cell types are involved in the structural and functional properties of the V-SVZ niche. Glial cells, such as astrocytes and microglia, have a major role, which has been recently reviewed elsewhere (Schneider et al., 2019; Sirerol-Piquer et al., 2019; Marchetti et al., 2020; Araki et al., 2021). In this review, we focus on the role of ependymal and vascular cells in the regulation of V-SVZ neurogenesis. After describing their contribution to the structure of the V-SVZ niche, we discuss the molecular signals mediating the effects of ependymal and vascular cells on NSPC proliferation and lineage progression, and their implication in the alterations of V-SVZ neurogenesis associated with neuropathological conditions.

## The Complex Microenvironment of the Ventricular-Subventricular Zone Niche

### Cytoarchitecture of the Adult Ventricular-Subventricular Zone

**FIGURE 1:**
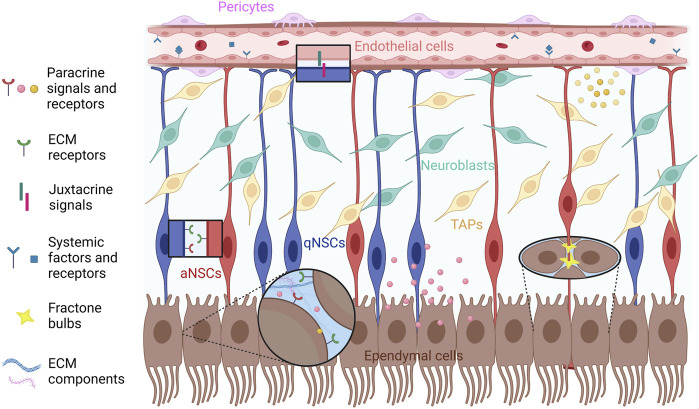
Schematic representation of the cell types of the adult V-SVZ niche that are specifically discussed in this review (qNSCs, aNSCs, TAPs, NBs, ependymal cells, endothelial cells and pericytes), and the different types of signals (systemic, paracrine, juxtacrine, ECM-associated) involved in the functional interactions among NSPCs, ependymal and vascular cells. The diagram does not include non-neurogenic astrocytes, oligodendrocyte precursors, microglia, neuronal cells and neuronal terminals, which are also present in the V-SVZ; moreover, it does not faithfully represent the relative location and distance from the apical and basal compartments of NSCs, TAPs and NBs. See text for further details. Created with BioRender.com.

The microenvironment of the V-SVZ niche is essential for the maintenance of the NSPC pool and the correct reception of signals that modulate NSPC activity and the levels of neurogenesis. The wide range of extracellular cues acting in the niche milieu and their complex interactions have been extensively investigated, but remain only partially understood. These signals originate in part from cell populations residing or projecting within the niche, which include the subpopulations of the neurogenic lineage (NSCs, TAPs, and NBs), different neuronal subtypes (such as cholinergic neurons and dopaminergic terminals) as well as non-neurogenic cell types (glial cells, ependymal cells and vascular cells). Another important part of these signals comes from distant sites and are transported into the niche through blood vessels. The V-SVZ has two major niche compartments that provide different cues: the apical ependymal layer, which faces the LV, and the vascular plexus on the opposite side. NSCs are generally located underneath the ependymal compartment; their apical processes extend through the ependymal layer to contact the cerebrospinal fluid (CSF) in the LV, and their basal processes make contact with the blood vessels (Figure 1). TAPs tend to lie closer to the vasculature than NSCs; however, NSC somas intercalated between ependymal cells or directly contacting the blood vessels have also been observed (Shen et al., 2008; Tavazoie et al., 2008). The V-SVZ niche also includes NBs, which form elongated clusters known as chains that converge at the level of the rostral migratory stream, a migratory path from the V-SVZ to the olfactory bulb (Lim and Alvarez-Buylla, 2016). The apical processes of groups of NSCs are surrounded by ependymal cells forming rosette structures similar to pinwheels (Mirzadeh et al., 2008; Codega et al., 2014). Ependymal cells are multi-ciliated cells that form the brain ventricular epithelium; among them, two subpopulations have been characterized: multi-ciliated ependymal cells (E1), which are predominant, and a rare subset of bi-ciliated ependymal cells (E2) (Mirzadeh et al., 2008). Astrocytes constitute another major component of the V-SVZ microenvironment, which provides nutrition and support to neurons, also acting as key mediators of the inflammatory response in various CNS diseases. Furthermore, by affecting NPSC proliferation and differentiation, astrocytes modulate neurogenesis or gliogenesis both in physiological conditions and in the injured CNS (Cassé et al., 2018; Schneider et al., 2019; Araki et al., 2021). Another functional component of the V-SVZ stem cell niche is the LV choroid plexus (LVCP), the main producer of CSF. The LVCP releases various signaling molecules in the CSF in response to physiological stimuli from the circulation, the nervous system, and the immune system. Secreted factors from the LVCP regulate multiple aspects of adult V-SVZ NSPC function (Silva-Vargas et al., 2016).

### Extracellular Matrix and Vascular System in the Adult Ventricular-Subventricular Zone

It is well known that NSCs require a specialized vascular niche, implying that NSPC regulation in the V-SVZ relies on cues provided by the local vasculature, which can include signals produced by the vascular cell populations (endothelial cells, pericytes, smooth muscle cells), the basement membrane (BM) of blood vessels and humoral factors transported in the bloodstream. The mouse V-SVZ niche contains a planar vascular plexus, with blood vessels making up 2.6% of the total V-SVZ volume. Confocal imaging shows that larger blood vessels run parallel to the ventricular surface underneath the ependymal layer and branch to form a dense network of vessels. This network extends throughout the length of the V-SVZ and is highly associated with the neurogenic lineage (Shen et al., 2008; Tavazoie et al., 2008). Of note, TAPs and aNSCs were found nearer to the vasculature than NBs, on average, suggesting that proximity to the blood vessels may help to maintain NSPC properties (Shen et al., 2008; Tavazoie et al., 2008). Blood vessels in the V-SVZ are largely capillaries, but dividing NSPCs were also found adjacent to larger vessels (arterioles and venules). Although the contacts between NSPC processes and blood vessels are difficult to study, many NSPC somas were observed adjacent to the vasculature; the majority of these contacts were located at specific points, where the vessels were devoid of astrocyte endfeet and/or pericytes (Shen et al., 2008; Tavazoie et al., 2008). Supporting a role for V-SVZ blood vessels in modulating NSC activity, experimentally induced stroke, which is known to stimulate V-SVZ neurogenesis in mice, caused an increase in blood vessels up to 5.7% of the total V-SVZ volume, mostly due to expanded capillaries, which was accompanied by an increase in the number of NSC processes interacting with the vasculature (Zhang et al., 2014).

BMs are specialized ECM sheets, which often underlie endothelial and epithelial layers. The ECM is ubiquitously found in the V-SVZ niche, and NSPCs interact with the ECM in several critical phases of the neurogenic process, such as NSPC division, adhesion and migration. The ECM is fundamental to cell-cell interactions and provides mechanical support to the resident cells in the V-SVZ niche. Furthermore, the ECM plays a crucial role in regulating the activity of V-SVZ cells by incorporating signaling molecules and binding growth factors and cytokines released in the niche environment (Kerever and Arikawa-Hirasawa, 2021). Among the ECM constituents, it is possible to find laminins, which are abundant around blood vessels in the V-SVZ niche. The integrin α6β1 heterodimer, which mediates the interaction with ECM laminins, is expressed in V-SVZ NSPCs, with low expression levels in NBs. Notably, antibodies blocking the binding of integrin α6β1 to laminins decreased the interaction of V-SVZ NSPCs with blood vessels and increased their proliferation, suggesting that the vasculature produces signals regulating NSPC activity (Shen et al., 2008). Disruption of integrin-laminin binding also led to abnormal NSPC adhesion and proliferation in the developing mouse cerebral cortex (Loulier et al., 2009). These observations highlight the different roles of the ECM in the regulation of the structural and neurogenic properties of the V-SVZ niche.

Besides the BMs of blood vessels, the V-SVZ contains extravascular BM structures known as fractones, which are abundantly distributed in the ependymal compartment of the V-SVZ, providing anchoring spots to the apical endfeet of NSCs, as well as acting as reservoirs for humoral factors (Nascimento et al., 2018; Sato et al., 2019; Kerever and Arikawa-Hirasawa, 2021). Two morphologically different fractone components have been described. The first component consists in thin fingerlike stems apparently emerging from capillaries, which branch profusely as they approach the ventricular surface; a second component is located in the ependymal layer and is made of small BM deposits with a speckled distribution in the LV wall, both on the apical and the basolateral sides of ependymal cells, which have been variously called hubs, bulbs or speckles (Figure 1) (McClenahan et al., 2016; Nascimento et al., 2018; Sato et al., 2019). These structures, which we will refer to as fractone bulbs from now on, were at first thought to correspond to the bulging terminations of individual stems, both emanating from the vascular BMs. Although the origin of fractone stems and their relation to fractone bulbs remains unclear, there is now increasing evidence that fractones, or at least their better characterized bulb component, are spatially separated from vascular BMs and chemically distinguishable from them (McClenahan et al., 2016; Nascimento et al., 2018; Sato et al., 2019). By staining for laminins to visualize fractone bulbs and β-catenin to visualize cell junctions in the ependymal layer, it was revealed that bulbs are preferentially located at the interface between neighboring cells in the ependymal layer. V-SVZ NSCs interact with BMs of blood vessels through their basal processes, while their cell bodies and apical processes contact several fractone bulbs at the same time. Furthermore, fractone bulbs are frequently located at the center of pinwheels formed by adjacent ependymal cells. This location, where NSC processes contact the CSF, coincides with NSC hubs from where several processes radially extend to interact with surrounding fractone bulbs. Three-dimensional reconstruction and orthogonal views of contact points between NSC processes and fractone bulbs at pinwheel centers showed direct contact between these two structures (McClenahan et al., 2016; Nascimento et al., 2018; Sato et al., 2019). Fractone bulbs, thus, have a structural role, providing an adherence spot for ependymal cell bodies and the apical processes of NSCs. In addition, the pinwheel center is a pivotal site for the regulation of NSC activity, since the primary cilium in the apical process can act as a signaling hub. The interaction between apical processes and fractone bulbs increases in complexity during aging, since tunneled bulbs envelop the apical NSC region, leading to increased fractone size in aged mice (Nascimento et al., 2018; Kerever and Arikawa-Hirasawa, 2021). Fractones contain collagen IV, several laminin isoforms, nidogen, and heparan sulfate proteoglycans (HSPGs), such as collagen XVIII, agrin and perlecan, similarly to other BMs. Although there are discrepancies in their reported composition in different studies, fractone bulbs appear to have molecular traits that distinguish them from vascular BMs, such as the presence of specific laminin isoforms, confirming the idea that the origin and function of these structures is distinct (Nascimento et al., 2018; Sato et al., 2019). Since many growth factors, cytokines and chemokines are heparin-binding molecules, heparan sulfates present in BMs can modulate their localization and binding to specific receptors, as shown for fibroblast growth factors (FGFs) and bone morphogenetic proteins (BMPs), which are implicated in V-SVZ NSPC regulation (Mercier, 2016; Kerever and Arikawa-Hirasawa, 2021; Marqués-Torrejón et al., 2021).

To further investigate what makes the environment of the neurogenic niche different from the non-neurogenic brain parenchyma, proteomic approaches have been recently used to compare the protein composition of the adult V-SVZ niche with that of the cerebral cortex gray matter. This analysis included an in depth investigation of ECM-associated and ECM core proteins, or matrisome (Kjell et al., 2020). Remarkably, the V-SVZ matrisome was more enriched in soluble ECM-associated proteins (such as S100 and serpin proteins) and less enriched in insoluble structural ECM proteins (such as collagens and laminins) in comparison with the cortical gray matter. Furthermore, several ECM-associated proteins and some ECM core proteins showed increased solubility in the V-SVZ. Surprisingly, the V-SVZ was stiffer than the cortical gray matter, possibly due to its enrichment for insoluble ECM proteins that are associated with tissue stiffness (such as laminin-b2, nidogen-1, and perlecan), and for the ECM cross-linker enzyme transglutaminase-2, which is expressed in ependymal cells and NSCs (Kjell et al., 2020). These results strongly suggest that a specific matrisome profile is pivotal to the unique properties of the V-SVZ extracellular environment.

Given the widespread importance that the correct reception of environmental stimuli by the niche has on tissue homeostasis and on the maintenance of the stem cell pool, the V-SVZ niche is expected to play a crucial role in the modulation of neurogenesis by external signals conveyed via the vasculature or the CSF. This modulation is possible thanks to the close communication of V-SVZ cells with the CSF and with the vascular system. Changes in these interactions can determine the onset of cognitive deficits during physiological and pathological aging. In the following sections, we will consider the complex crosstalk between NSPCs and specific non-neurogenic components of the V-SVZ niche, trying to elucidate how this influences NPSC function and neurogenesis.

## A Constant Crosstalk With the Non-Neural Components of the Ventricular-Subventricular Zone Niche Regulates Neural Stem/Progenitor Cell Quiescence, Proliferation and Lineage Progression: The Role of Ependymal and Vascular Cells

### Molecular Signatures of Neural Stem Cells Reveal Extensive Interactions With the Ventricular-Subventricular Zone Niche Environment

**FIGURE 2:**
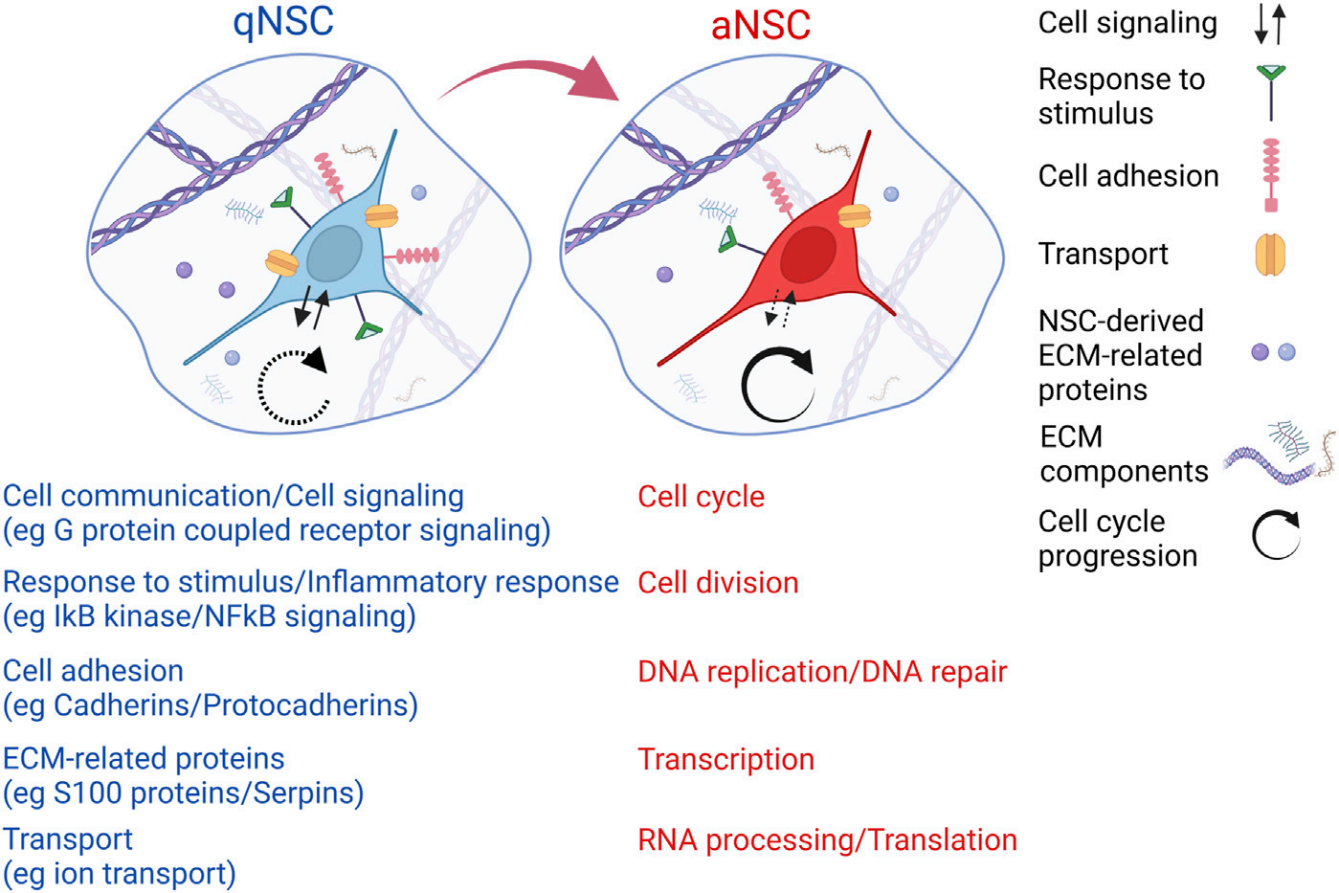
Schematic representation of some of the molecular changes that may happen during NSC activation, as suggested by transcriptomic comparison of qNSCs and aNSCs of the adult V-SVZ (Codega et al., 2014; Morizur et al., 2018; Kjell et al., 2020; Xie et al., 2020; Belenguer et al., 2021). In particular, molecular profiles related to cell communication/signaling, response to stimulus, cell adhesion, transport and ECM decrease during NSC activation (red color, examples of each category are indicated in brackets); profiles related to cell cycle, cell division, DNA replication/repair, transcription, RNA processing/translation increase during NSC activation (blue color). The diagram does not include additional functional categories that show activation-related changes in NSCs according to published studies. See text for further details. Created with BioRender.com.

Mouse adult V-SVZ NSCs share a common cell lineage with embryonic telencephalic RGCs, but differ from them in terms of their largely quiescent status and of the neuronal subtypes generated by their progeny (Fuentealba et al., 2015; Furutachi et al., 2015). The functional properties that distinguish adult NSCs from embryonic RGCs might be due to an intrinsic change in cell identity taking place during the transition from RGCs to B1 cells, or to different extrinsic cues acting in the developing and in the adult V-SVZ niche. To address this question, single-cell RNA sequencing has been employed to profile the transcriptome of NSPC populations of the developing cerebral cortex from embryonic day 11.5 (E11.5) until E17.5 and of the postnatal V-SVZ from P6 until adulthood (P61) (Yuzwa et al., 2017; Borrett et al., 2020). This analysis has revealed a core transcriptional signature linked to NSC identity, which is shared between embryonic RGCs and adult V-SVZ NSCs. Furthermore, many of the differentially expressed genes between adult NSCs and embryonic RGCs showed similar transcriptional changes when comparing adult NSCs and TAPs, indicating that adult NSC activation and progression along the neurogenic lineage involves the awakening of developmental programs that operate in embryonic RGCs. These observations suggest that the different behavior of V-SVZ NSCs as compared with embryonic RGCs is not intrinsically determined, but is orchestrated by the extrinsic signals of the V-SVZ niche impinging on NSCs. Of note, several genes upregulated in the transition from embryonic RGCs to adult NSCs were related to the interaction with cues present in the niche environment, such as neurotransmitters, ions, receptor ligands and ECM proteoglycans (Borrett et al., 2020). Confirming the importance of extracellular niche cues in the regulation of NSC fate, molecular profiling of V-SVZ NSCs of different ages (young adult vs aged NSCs) or in different activation states (qNSCs vs aNSCs) has shown that functional modifications in NSC activity are linked to transcriptomic changes in pathways mediating NSC response to the niche milieu (Figure 2). In particular, an enrichment of gene categories related to cell signaling, cell communication, response to stimulus, cell adhesion, transport, and ECM has been observed among the gene sets preferentially expressed in qNSCs in comparison with aNSCs (Codega et al., 2014; Morizur et al., 2018; Kalamakis et al., 2019; Kjell et al., 2020; Belenguer et al., 2021). Of note, gene categories related to the inflammatory response were enriched among the upregulated genes in aged NSPCs or in qNSCs as compared to young adult NSPCs or to aNSCs (Dulken et al., 2019; Kalamakis et al., 2019; Belenguer et al., 2021). Functional experiments with candidate signaling intermediates identified through these studies support a causative role of the molecular changes related to NSC-niche interactions in the cellular modifications observed during NSC activation or aging (Codega et al., 2014; Morizur et al., 2018; Kalamakis et al., 2019; Belenguer et al., 2021). Altogether, these observations suggest that extracellular niche cues, and the molecular programs dictating how NSCs sense these cues and respond to them, are instrumental in orchestrating NSC decisions. In the following sections, we will specifically focus on the role of signals originating from niche ependymal and vascular cells in the regulation of V-SVZ neurogenesis.

### Interactions Among Ependymal Cells, Neural Stem/Progenitor Cells and the Extracellular Matrix Regulate Postnatal Development and Adult Neurogenesis in the Ventricular-Subventricular Zone Niche

Over the years, many studies have analyzed the role of different cellular components of the adult V-SVZ niche in NSC regulation. As described above, ependymal cells are an important cellular constituent of the adult V-SVZ. In the mouse V-SVZ, ependymal cell maturation takes place during the first 3 weeks of postnatal development; during this time, ependymal cells upregulate the expression of the Foxj1 transcription factor and modify their apical surface, which expands and becomes multiciliated. Moreover, they contribute to the deposition and coalescence of extravascular ECM aggregates, or hubs, on the ventricular surface, likely coinciding with the apically-located fractone bulbs or speckles described in the adult V-SVZ (McClenahan et al., 2016; Nascimento et al., 2018; Sato et al., 2019). Ependymal maturation and ECM hub formation are accompanied by the emergence of the pinwheel organization of the ependymal layer; several ependymal cells cluster around the apical processes of the emerging B1 cells, with EMC hubs relocating at the interface between ependymal cells and B1 cells at the center of pinwheels. These processes are largely completed by postnatal day 21 (P21) (McClenahan et al., 2016). Although they were previously thought to be quiescent cells endowed with a latent neurogenic potential in response to specific stimuli, recent studies have used finer *in vivo* labeling techniques to distinguish ependymal cells from V-SVZ NSCs, finding no evidence of ependymal cell mitosis, cell loss, or migration following brain injury (Shah et al., 2018). Single-cell RNA sequencing analysis of sorted ependymal and NSPC populations has shown a significant overlap of the transcriptomic profiles of ependymal cells and qNSCs (in agreement with their shared developmental lineage), although the former cell type is characterized by the expression of a cohort of genes related to cilia that distinguish them from NSPCs (Shah et al., 2018).

The simultaneous emergence of ependymal cells, B1 cells and ECM hubs connecting them during postnatal development makes it very likely that extracellular signals mediating a crosstalk among these components play a crucial role in the establishment of the V-SVZ niche. Confirming this idea, dystroglycan (DAG), a transmembrane ECM receptor, is required for proper V-SVZ development. DAG is expressed in RGCs and, at higher levels, in maturing ependymal cells, where it colocalizes with extravascular ECM hubs. In mice, conditional DAG deletion in RGCs and their progeny (DAG cKO) caused a reduction in the number of ECM hubs, delayed ependymal cell maturation and led to smaller and disorganized pinwheels at P21. These effects could be recapitulated in the context of *in vitro* ependymal differentiation of P0 SVZ cells in the presence DAG-blocking antibodies; co-treatment with Notch pathway inhibitors restored the formation of Foxj1-positive multiciliated ependymal cells, but not their clustering into pinwheel-like structures, indicating that DAG regulates ependymal maturation via Notch inhibition and pinwheel formation through a different mechanism. DAG cKO also caused an abnormal increase of RGC proliferation and oligodendrocyte precursor formation in the postnatal V-SVZ, showing that DAG-dependent signaling controls both the structural and functional development of this niche (McClenahan et al., 2016).

Matrix metalloproteinase 12 (MMP12) is another molecule upregulated in developing ependymal cells, which is required for their maturation, as well as the proper formation of ECM hubs and pinwheels in the postnatal V-SVZ. Remarkably, MMP12 has both extracellular and intracellular roles in V-SVZ development. These roles have been dissected thanks to MMP12 mutant mice specifically lacking secreted MMP12, in which intracellular MMP12 (icMMP12) has been depleted by RNA interference. This work has shown that icMMP12, but not secreted MMP12, is involved in the maturation of multiciliated Foxj1-positive ependymal cells, whereas the lack of secreted MMP12 caused a reduction of ECM hubs and an increase of the NSC/pinwheel ratio. MMP12 dysfunction also led to increased NSC activation at P7, indicating that, directly (via MMP12 uptake in NSCs) or indirectly, this protein promotes NSC quiescence in the postnatal SVZ (Shan et al., 2018).

In the postnatal V-SVZ, developing ependymal cells, along with the LVCP, also produce Tsukushi (TSK), a secreted leucine-rich proteoglycan implicated in the extracellular modulation of several signaling pathways associated with neurogenesis, such as TGFβ, Notch and Wnt, through direct binding to ligands or receptors for these pathways (Ohta et al., 2004; Kuriyama et al., 2006; Ohta et al., 2011). During postnatal development, the functional activity of the V-SVZ niche is dysregulated in TSK-deficient mice, as shown by an early increase in NSPC proliferation, which is followed by an abnormal rise in apoptotic cells (Ito et al., 2021). Altogether, these studies suggest that extracellular molecules released by ependymal cells in the niche environment, along with receptors mediating the interaction between ependymal cells and the ECM, are instrumental in orchestrating the correct assembly of the V-SVZ niche and to regulate NSPC activity during the transition from embryonic RGCs to adult B1 cells.

**FIGURE 3:**
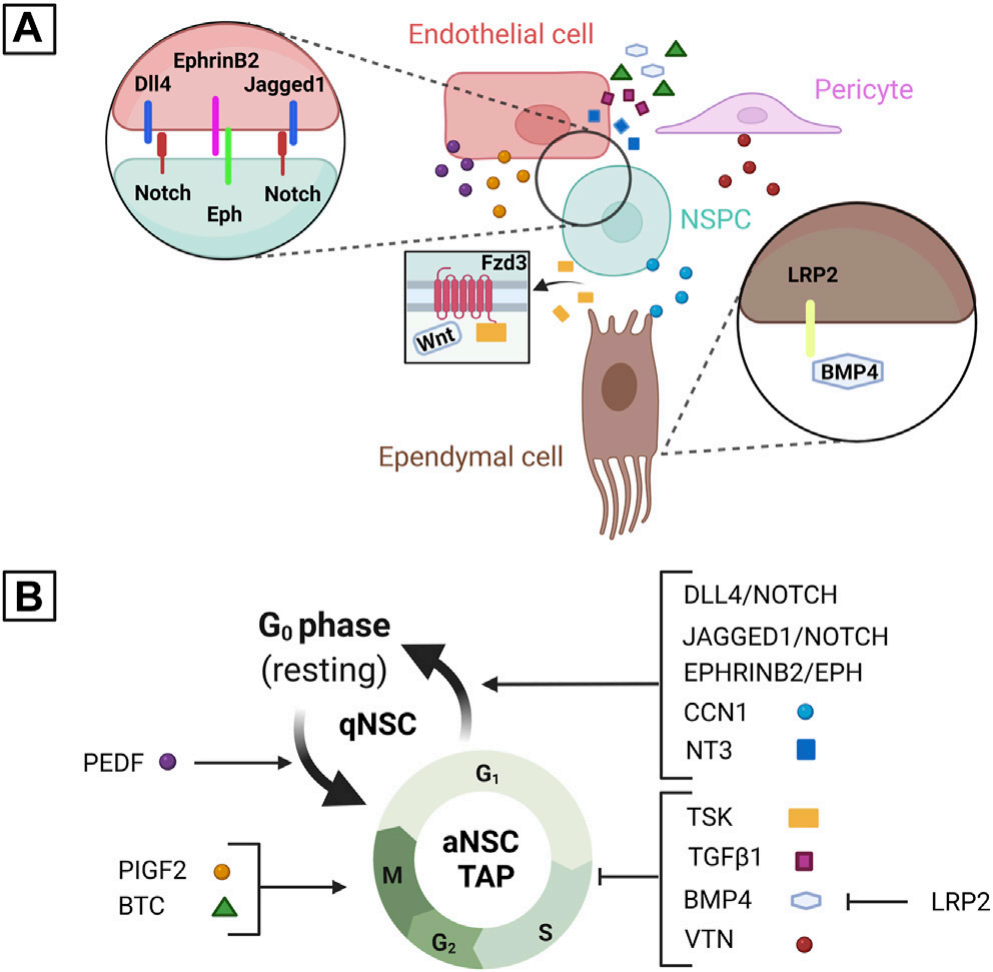
Schematic representation of some of the juxtacrine and paracrine signals mediating the regulation of V-SVZ NSPCs by ependymal and vascular cells. **(A)** shows some of the signals and receptors produced by each cell type (endothelial cells, pericytes, ependymal cells and NSPCs). **(B)** shows the role of each signal in the regulation of NSC activation or NSPC proliferation based on the studies described in this review. In particular, CCN1, Dll4, Jagged1, ephrinB2, and NT3 are involved in promoting NSC quiescence, whereas PEDF is involved in promoting NSC activation; PlGF2 and BTC are involved in promoting NSPC proliferation, whereas TSK, TGFβ1, BMP4, and VTN are involved in inhibiting it. LRP2 is involved in promoting NSPC proliferation indirectly by inhibiting BMP4 activity. TSK may affect NSPC proliferation by binding Fzd3, a Wnt receptor, and modulating Wnt signaling. Please note that some of these molecules may play multiple roles in the regulation of NSC activation/quiescence and of NSPC proliferation that are not represented in this diagram. See text for further details. Created with BioRender.com.

In the adult V-SVZ, ependymal cells continue to act as a source of niche factors modulating NSC function thanks to their close contact with B1 cells along the ventricular surface (Figure 3). Recent studies indicate that some of these signals may be important to restrict the expansion of the adult V-SVZ NSC pool, which may help to maintain its long-term neurogenic capacity. In particular, the secreted ECM-associated protein CCN1 (cellular communication network factor 1), which is involved in the regulation of cell proliferation by interacting with integrins, is specifically expressed in ependymal cells in the adult mouse V-SVZ (Wu et al., 2020). Conditional CCN1 inactivation in these cells caused transient NSC expansion, leading to an increase in the density of NSCs and pinwheel units that persisted in aged mice. These effects were accompanied by an increase of aNSCs and a corresponding decrease of TAPs and newborn neurons, indicating that CCN1 loss promotes NSC self-renewal at the expense of lineage progression. Four weeks after CCN1 deletion, however, the amounts of aNSCs, TAPs and newborn neurons were similar to those present in control mice. This suggests that CCN1-deficient NSCs rapidly returned to quiescence after their initial activation and expansion, although the larger NSC pool of the CCN1-deficient V-SVZ conferred stronger regeneration capacity upon pharmacological depletion of the proliferating NSPC population (Wu et al., 2020). To elucidate the signaling pathways mediating the effects of ependymal CCN1 inactivation on NSCs, Wu and colleagues performed treatments with pharmacological inhibitors of epidermal growth factor receptor (EGFR) or with blocking antibodies against integrin α6β1. These proteins are expressed in V-SVZ NSCs, where they are involved either in NSC proliferation (EGFR) (Cochard et al., 2021), or in CCN1 binding (integrin α6β1) (Wu et al., 2020). Although integrin blocking antibodies had no effects on the NSC pool, EGFR inhibition prevented its expansion in the CCN1-deficient V-SVZ. Therefore, the ependymal factor CCN1, acting via EGFR signaling but not integrin α6β1, may help to control the size of the NSC reservoir in the adult V-SVZ by balancing NSC activation, self-renewal and differentiation.

BMPs are extracellular signals playing pivotal roles in the regulation of NSC proliferation in the adult V-SVZ (Joppé et al., 2015). In the developing neural tube, BMPs are modulated by the low-density lipoprotein receptor-related protein 2 (LRP2), which is expressed on the apical surface of the embryonic neuroepithelium. LRP2 expression persists in the ependymal cells of the V-SVZ and its loss causes a reduction in the proliferative capacity of this niche. This coincides with an increased expression of BMP2/4 proteins and of the BMP signaling components phospho-Smad1/5/8 and Id3 in the V-SVZ of adult LRP2-deficient mice. These results suggest that LRP2 acts in ependymal cells as a negative modulator of BMP signaling to promote adult V-SVZ neurogenesis (Gajera et al., 2010), possibly in collaboration with extracellular BMP antagonists secreted by ependymal cells (Lim et al., 2000). Ependymal cells also produce laminin α5, a major laminin component of fractone bulbs in the V-SVZ (Nascimento et al., 2018). Conditional deletion of the *Lama5* gene in ependymal cells, which causes nearly complete depletion of laminin α5 in fractone bulbs, leads to a reduction in the number of slowly dividing NSCs, and a concomitant increase in the number of mitotic NSPCs, when compared with control mice, suggesting that ependyma-derived laminin α5 in the ECM of V-SVZ fractone bulbs is required to maintain the NSC pool by constraining its activation or lineage progression (Nascimento et al., 2018). Another study, however, has pointed to V-SVZ NSCs as another important source of fractone-localized laminin α5, due to its depletion following conditional deletion of the *Lama5* gene in these cells; in the same study, expression of a laminin γ1 variant that cannot bind integrins in V-SVZ NSCs led to a reduction in fractone bulb number and size, in the contact between NSCs and fractone bulbs, and in NSC ability to form neurospheres *in vitro* (Sato et al., 2019). Fractones also contain HSPGs, such as perlecan, which can interact with extracellular signals present in the V-SVZ milieu, such as FGF2 and BMPs, and modulate signal transduction through their receptors (Kerever et al., 2014; Mercier and Douet, 2014). The heparan sulfate composition of fractones changes with age, in parallel to the age-related neurogenic decline of the V-SVZ; although FGF2 binding to FGF receptors is not apparently affected by aging, alterations to FGF signal transduction in the aged V-SVZ have been reported (Yamada et al., 2017). Furthermore, the V-SVZ of perlecan-deficient mice showed decreased NSPC proliferation and neurogenesis, whereas the lack of enzymes catalyzing HSPG desulfation, which are produced by neurons in the adjacent brain parenchyma and by the LVCP, stimulated V-SVZ neurogenesis (Kerever et al., 2014; Kerever et al., 2021). Taken together, the results of these studies indicate that both laminins and HSPGs are functionally important fractone components, which regulate the neurogenic activity of the V-SVZ by mediating the interactions of NSCs with the niche environment. More work will be needed to clarify the contribution of different niche cell types to the chemical composition of fractones and the role of different fractone components in the modulation of V-SVZ neurogenesis.

### Extracellular Signals Produced by Endothelial Cells and Pericytes as Key Regulators of Adult Ventricular-Subventricular Zone Neurogenesis

An important characteristic of the V-SVZ niche is the close contact of NSPCs with vascular cells. This contact is facilitated by a permeable blood-brain barrier, which is present in the V-SVZ , but not in the subgranular zone hippocampal niche (Lin et al., 2019). Therefore, the vasculature of the V-SVZ could potentially modulate neurogenesis by providing an access route for systemic factors to this niche, and/or by means of contact-dependent or paracrine signals provided by vascular cell types, such as endothelial cells and pericytes. Experimentally-induced stroke in rodents is a useful paradigm to address the role of the V-SVZ vascular system on neurogenesis, since it acts as a strong angiogenic and neurogenic stimulus in this niche (Chen et al., 2005; Thored et al., 2006). Stroke leads to an increase of growth factors and cytokines in the blood, including VEGF (Zhang et al., 2000; Jin et al., 2002). Although VEGF-A infusions in the bloodstream of adult mice can promote V-SVZ neurogenesis, its main receptor, VEGFR2, is expressed by endothelial cells in the V-SVZ, but not by NSPCs, either in physiological conditions or after stroke (Lin et al., 2019); this suggests an indirect effect of systemic VEGF-A on NSPC activity, differently from the proposed direct modulation of V-SVZ neurogenesis by VEGF-C acting through VEGFR3 (Calvo et al., 2011). Supporting this hypothesis, stroke caused an upregulation of the Notch ligand Dll4 in endothelial cells and pericytes of V-SVZ blood vessels *in vivo*, and VEGF treatments promoted Dll4 expression in endothelial cell cultures *in vitro*. Dll4 upregulation in V-SVZ vascular cells after stroke was accompanied by Notch activation in adjacent proliferating NSPCs and NBs (Lin et al., 2019). Although the role of Dll4 was not directly addressed in this study, these results suggest that endothelial cells may regulate V-SVZ neurogenesis through the modulation of Notch signaling in NSPCs.

Confirming an important role of the endothelium in the extrinsic regulation of NSPC activity in the V-SVZ, several studies have shown that NSC activation and NSPC proliferation *in vitro* are affected by co-culture with endothelial cells or by exposure to endothelial cell conditioned media (Mathieu et al., 2008; Ottone et al., 2014; Crouch et al., 2015; Bicker et al., 2017). Of note, the former treatment inhibits NSPC proliferation, whereas the latter treatment stimulates it, suggesting that endothelial cells produce both contact-dependent and paracrine signals, which can exert different effects on NSPCs. Notch and ephrin juxtacrine signaling pathways play a role in mediating the contact-dependent modulation of NSPC activity by endothelial cells. In particular, stimulation of Notch signaling in V-SVZ NSCs by Dll4 expressed by endothelial cells appears to inhibit NSC activation, as shown by the increase of proliferating aNSCs following conditional Dll4 deletion in endothelial cells and their decrease following LV injection of an adenovirus encoding for Dll4. Highlighting the complex crosstalk between NSPCs and vascular cells in the V-SVZ, both these cell types secrete the EGFL7 protein, which binds to the extracellular region of Notch, promoting the interaction between Dll4 and Notch, Notch pathway activation and NSC quiescence (Bicker et al., 2017). V-SVZ endothelial cells also express Jagged1, another Notch ligand, and ephrinB2, which is a ligand for Eph receptors; in NSPCs, Jagged1 activates the Notch pathway and its target genes, ephrinB2 inhibits ERK activity and cyclinD/E expression. Conditional deletion of Jagged1 or ephrinB2 in endothelial cells caused an increase of aNSCs after a few days, followed by a decrease of the total NSC population a few weeks later, with clear additive effects in mice deficient for both proteins. Therefore, these endothelial juxtacrine signals collaborate through distinct signaling pathways to maintain the V-SVZ NSC pool by promoting NSC quiescence (Ottone et al., 2014). Another Notch ligand, Dll1, is expressed in V-SVZ aNSCs and TAPs. Upon aNSC division, Dll1 asymmetrically segregates to one daughter cell and may activate Notch signaling in its sister cell, promoting a quiescent state (Kawaguchi et al., 2013). Thus, coordinated Notch modulation by ligands expressed in NSPCs or endothelial cells may be important to regulate the transition between qNSC and aNSC states.

Although contact-dependent signals expressed by endothelial cells inhibit NSC activation, these cells also produce soluble signals that can stimulate NSPC proliferation, as shown by *in vitro* treatments of V-SVZ NSPCs with conditioned media from endothelial cultures; one of these signals has been identified as placental growth factor 2 (PlGF2), which is secreted by V-SVZ endothelial cells and promotes NSC activation and NSPC proliferation through VEGFR-dependent signaling. Unexpectedly, endothelial cells of the adult cerebral cortex produce higher levels of PlGF2 and support NSPC proliferation more efficiently than V-SVZ endothelium, although the adult cortex is a non-neurogenic region (Crouch et al., 2015). Two additional secreted proteins that are produced by endothelial cells in the V-SVZ and stimulate NSPC activity are betacellulin (BTC), which is also expressed in the LVCP, and pigment epithelial-derived factor (PEDF), which is also expressed in ependymal cells. BTC is a member of the EGF family, acting via both EGFR and ErbB4 receptors, which can support NSC self-renewal and NSPC proliferation *in vitro* similarly to EGF, although EGF activity is specifically mediated by EGFR (Gómez-Gaviro et al., 2012). PEDF is a member of the serine protease inhibitor superfamily, which can promote NSC self-renewal *in vitro* in combination with EGF, but is unable to support NSPC proliferation on its own (Ramírez-Castillejo et al., 2006). Infusions of BTC or BTC-blocking antibodies into the LV promote or repress adult V-SVZ neurogenesis, respectively, by increasing or decreasing the amount of proliferating NSPCs, which may involve the modulation of both NSC activation and TAP/NB proliferation (Gómez-Gaviro et al., 2012). Similar experiments performed with PEDF, or a C-terminal fragment of this protein acting as a competitive inhibitor of its activity, also modulate V-SVZ neurogenesis, but PEDF appears to specifically promote NSC activation, with little, if any effects on TAP/NB proliferation (Ramírez-Castillejo et al., 2006).

Surprisingly, endothelial cells also express paracrine factors that inhibit NSPC proliferation, such as the neurotrophic factor NT3 (Delgado et al., 2014), and the TGFβ superfamily proteins BMP4 and TGFβ1 (Pineda et al., 2013; Daynac et al., 2014; Marqués-Torrejón et al., 2021). NT3 is produced in endothelial cells of the V-SVZ and accumulates in the CSF via the LVCP; NT3 protein from the blood vessels and/or the CSF is received by V-SVZ NSCs, which respond by increasing nitric oxide synthase (NOS) activity, leading to cell cycle inhibition. Supporting this sequence of events, mice with heterozygous *NT3* inactivation or with conditional deletion of *NT3* in endothelial cells showed increased numbers of aNSCs in the 2 months old V-SVZ. Moreover, injection of NT3 protein in the LV caused a decrease of aNSCs in control mice, but not in mice lacking NOS3, the NOS isoform expressed in V-SVZ NSCs. Furthermore, *in vitro* treatments with NT3 protein, or with conditioned media from control or NT3-deficient endothelial cells, indicated that endothelial NT3 can stimulate NOS3 activity in NSCs. The number of BrdU-retaining NSCs was decreased in the V-SVZ of 8 months old NT3 mutant or NOS3 mutant mice, suggesting that the NT3-NOS3 axis is necessary to preserve the pool of V-SVZ NSCs from exhaustion by limiting their activation (Delgado et al., 2014).

In other studies, extracellular BMP antagonists, such as Noggin, or knockdown of Smad5, a BMP signaling component, were shown to restore the proliferation of NSPCs co-cultured with endothelial cells (Mathieu et al., 2008); moreover, *in vivo* treatments with anti-TGFβ1 antibodies or with TGFβ1 receptor inhibitors partially rescued NSPC proliferation and V-SVZ neurogenesis in aged mice (Pineda et al., 2013; Daynac et al., 2014), suggesting that endothelial-derived TGFβ superfamily signals can inhibit NSPC cell cycle. It should be noted, however, that the endothelial cultures used to study the role of BMP signaling were not V-SVZ-specific, and that TGFβ1 pathway inhibition did not improve NSPC proliferation in young adult mice, in agreement with the low expression levels of TGFβ1 in the V-SVZ blood vessels of these mice (Pineda et al., 2013).

Endothelial cells also release factors modulating the spatial association of NSPCs with the vascular microenvironment, such as stromal-derived factor 1 (SDF1, also known as CXCL12) (Zhu et al., 2019). SDF1 is specifically expressed in endothelial cells of V-SVZ capillaries, but not in the endothelium of larger vessels (arterioles and venules) or in pericytes. SDF1 receptor, CXCR4, is expressed in the neurogenic lineage, with higher transcript levels in aNSCs and TAPs, and lower levels in NBs. Proliferating NSPCs are preferentially, though not exclusively, associated with SDF1-expressing vessels, whereas slowly dividing NSCs tend to locate closer to SDF1-negative vessels; supporting a causal link between SDF1 expression and NSPC localization, conditional deletion of CXCR4 in NSPCs led to increased detachment of proliferating NSPCs from SDF1-expressing vessels. Impaired SDF1 signaling also caused a short-term increase of proliferating NSPCs (possibly due to an increased transition from aNSCs to TAPs), followed by their depletion after a few weeks. These results suggest that aNSC proximity to blood vessels, which is stimulated by SDF1, may help to maintain the proliferating NSPC pool by limiting aNSC progression along the neurogenic lineage. Notably, aging is associated with CXCR4 downregulation and increased detachment from SDF1-expressing vessels of proliferating NSPCs, in agreement with their depletion in the aged V-SVZ (Zhu et al., 2019). Altogether, these studies indicate that endothelial cells play a complex role in the regulation of V-SVZ neurogenesis, producing different types of signals that can exert collaborative or antagonistic effects on NSPC activity. Remarkably, the molecular cues provided by the V-SVZ endothelium or their activity can be influenced by various extrinsic agents, such as systemic blood factors, hypoxia and aging (Pineda et al., 2013; Lin et al., 2019), as well as signals from other V-SVZ cell populations, such as Noggin from ependymal cells and astrocytes (Lim et al., 2000; Peretto et al., 2004), and EGFL7 from NSPCs (Bicker et al., 2017). This suggests that changes in the signaling activity of endothelial cells may play an important role in the modulation of adult V-SVZ neurogenesis by external stimuli, aging or disease.

A role in the regulation of adult neurogenesis was also found for pericytes present in the V-SVZ niche, since treatments of V-SVZ NSPCs with conditioned media from pericyte cultures derived from the adult mouse V-SVZ stimulated NSPC proliferation, albeit less efficiently than media from endothelial cells, and increased their competence towards neuronal differentiation even more efficiently than endothelial media (Crouch et al., 2015). Vitronectin, a glycoprotein present in the blood and in the ECM and a binding partner of integrins, is strongly and specifically expressed by a subpopulation of V-SVZ pericytes. Furthermore, neurogenesis was enhanced in the V-SVZ of adult VTN-deficient mice, as shown by the increased number of proliferating NBs (Jia et al., 2019). At the molecular level, VTN promotes the expression of ciliary neurotrophic factor (CNTF), interleukin 6 (IL6) and leukemia inhibitory factor (LIF), since the expression of the genes coding for these proteins was upregulated in the adult V-SVZ by injections of VTN protein near this niche and downregulated in VTN mutant mice. VTN appears to stimulate CNTF expression in adjacent V-SVZ astrocytes, since it was upregulated in the V-SVZ of mice with conditional astrocyte-specific deletion of focal adhesion kinase, an important mediator of integrin-dependent signaling. The expression of IL6 and LIF was not altered in these mutants, suggesting that pericyte-derived VTN stimulates the production of these proteins in other V-SVZ cell types through a different molecular pathway. This pathway involves the gp130 receptor, since IL6 and LIF expression was upregulated less efficiently in the V-SVZ of mice co-injected with VTN and a gp130 inhibitor when compared with VTN injection alone. Notably, VTN injections repressed V-SVZ neurogenesis on their own, but promoted it together with gp130 inhibition (Jia et al., 2019). These data reinforce the idea that NSC activation and NSPC proliferation in the adult V-SVZ are modulated by a finely tuned equilibrium among the pro- and anti-neurogenic activities of several extracellular signals, in which endothelial cells and pericytes play a major role (Figure 3).

### Modulation of Adult Ventricular-Subventricular Zone Neurogenesis by Lateral Ventricle Choroid Plexus-Derived Cerebrospinal Fluid Signals

The CP is a highly vascularized epithelial tissue located within all brain ventricles, including the LV, which is largely responsible for CSF production. Although the LVCP is not a structural element of the V-SVZ, it acts as an essential functional component of this niche by releasing signaling molecules that reach the V-SVZ via the CSF. This role has been demonstrated by the ability of conditioned medium from adult mouse LVCP explants (LVCPcm) to promote NSPC proliferation both in V-SVZ cell cultures and following infusion in the LV of adult mice (Silva-Vargas et al., 2016). These results mimic the effects of treating NSPC cultures with CSF-supplemented media, suggesting that the LVCP is an important source of the CSF signals that regulate V-SVZ NSPCs (Silva-Vargas et al., 2016; de Sonnaville et al., 2020). In agreement with this idea, some of the ependymal and endothelial signals modulating mouse V-SVZ neurogenesis that we have described in previous sections, such as TSK, BTC, NT3, and SDF1, are also produced by the LVCP (Gómez-Gaviro et al., 2012; Silva-Vargas et al., 2016; Ito et al., 2021). Furthermore, transcriptomic and proteomic analysis of LVCP cells and of LVCPcm revealed that the LVCP secretes a variety of chemokines, growth factors and ECM-related proteins; several of them are known regulators of V-SVZ neurogenesis (eg FGF2, VEGF-A), or were able to modulate NSPC proliferation *in vitro* (eg TGFβ2, CXCL16) (Silva-Vargas et al., 2016). Remarkably, both *in vitro* and *in vivo*, LVCPcm from young adult mice could promote the proliferation of aged V-SVZ NSCs, whereas LVCPcm from aged mice repressed young adult NSC proliferation, when compared with age-matched treatments. Several signaling molecules were found to be differentially expressed in the young adult and in the aged LVCP; these included BMP5 and IGF1 (insulin growth factor 1), which were downregulated in aged samples and promoted NSPC proliferation *in vitro* (Silva-Vargas et al., 2016). These results are consistent with other studies reporting differences in the composition and in the effects on V-SVZ neurogenesis of CSF of different ages (Alonso et al., 2017; Bueno et al., 2020). The regulation of V-SVZ neurogenesis by LVCP-derived signals involves additional mechanisms that are not mediated by extracellular ligands. In particular, micro-RNA 204 (miR-204) is highly expressed in the LVCP of adult mice and released into the CSF within extracellular vesicles. Through this route, miR-204 may reach V-SVZ NSCs and inhibit the translation of several regulators of V-SVZ neurogenesis (eg Dlx1/2, Meis2, Sox11), which are involved in NSC activation and progression along the neurogenic lineage. Thus, LVCP-derived miR-204 may be important to modulate the NSC pool and the neurogenic output of the V-SVZ niche by means of a post-transcriptional mechanism. Supporting this role, inhibition of miR-204 function by antagomir injection into the LV or by expression of miR-204-specific tough decoy constructs in the LVCP reduced the NSC population and increased their activation and differentiation (Lepko et al., 2019). The transcription factor Otx2 is another LVCP-derived molecule implicated in the modulation of adult V-SVZ neurogenesis through its release in the CSF, although its target cells seem to be ependymal cells and non-neurogenic astrocytes rather than NSPCs (Planques et al., 2019). Altogether, these studies indicate that signals originating from the LVCP have a crucial role in the regulation of the V-SVZ niche and its age-related modifications. Given that the apical processes of V-SVZ NSCs are simultaneously exposed to molecules produced by ependymal cells and by the LVCP, the interactions between ependyma-derived and LVCP-derived signals in physiological and pathological conditions represent an interesting topic for future research.

## Alterations in the Crosstalk Between Ventricular-Subventricular Zone Ependymal Cells and Neural Stem/Progenitor Cells in Pathological Conditions

The elucidation of the molecular signals underlying the functional interactions among NSPCs and other V-SVZ cell populations is allowing the identification of an increasing number of molecules that are involved both in the regulation of adult neurogenesis and in human disease. This can have important implications for translational medicine in two ways; on the one hand, a deeper knowledge of the molecular mechanisms regulating V-SVZ neurogenesis may be harnessed to modulate this process in pathological conditions for therapeutic purposes; on the other hand, unraveling the mechanisms of action of disease-causing molecules in the context of V-SVZ neurogenesis may help to develop therapeutic interventions for different pathological processes that are affected by the same molecule. As an example of the potential therapeutic value of addressing the regulatory mechanisms of V-SVZ neurogenesis, we discuss here recent studies that have allowed to gain insight into the etiology of hydrocephalus through the characterization of signals mediating the crosstalk between ependymal cells and NSPCs in the V-SVZ niche.

The enlargement of the cerebral ventricles (ventriculomegaly) is a common structural finding in multiple developmental neuropsychiatric disorders including autism (Styner et al., 2005), which is often associated with cortical malformations such as microcephaly (Mishra-Gorur et al., 2014). Thus, ventriculomegaly is a convergent structural correlate of common neurodevelopmental disorders, suggesting that a dysregulation of the cell populations comprising the ventricular neuroepithelium may represent a unifying developmental condition underlying the ventricular expansion associated with various pathological contexts, including pediatric hydrocephalus. Human hydrocephalus is a common medical condition that is characterized by an enlargement of the cerebral ventricles and has been attributed to abnormalities in the flow or resorption of CSF. Hydrocephalus is classified into communicating and non-communicating forms, based on the absence or presence of structural blockage of the CSF flow (Jiménez et al., 2001). The CSF flow tract is a dynamic circulatory system that supplies the brain with essential nutrients and growth factors throughout development and during adulthood. The major cause for non-communicating hydrocephalus is the disrupted structural integrity of the ventricular system, whereas communicating hydrocephalus is due to a defective CSF flow. As current therapies rely on invasive procedures that are associated with high failure and complication rates, the identification of the molecular mechanisms underlying congenital hydrocephalus is a high priority for the treatment of this disease.

Ependymal cells, derived from RGCs, line the aqueduct and cerebral ventricular surface of mammalian species, make up the CSF tract and allow proper CSF flow. The failure of the normal generation, maturation and integrity of ependymal cells can cause early onset fetal hydrocephalus through aqueductal stenosis (Jiménez et al., 2001). In fact, aqueductal stenosis is the major contributing factor in congenital hydrocephalus, with a prevalence of 0.1%–0.3% of all live births (Carter et al., 2012). Moreover, abnormal ciliary beating by the multiciliated ependyma reduces CSF circulation, which may contribute to pathological accumulation of CSF and ventricular expansion. Mature ependymal cells, however, do not cover the majority of the LV wall until several days after birth in mice and humans (Coletti et al., 2018). Thus, NSPCs, rather than ependyma, are the primary constituent of the neuroepithelium lining the ventricular system during prenatal and early postnatal brain development. Several studies suggested that dysfunctional motile cilia may not be the primary cause of congenital hydrocephalus, as evidenced by ventricular expansion occurring before the development of motile cilia (Banizs et al., 2005; Carter et al., 2012). Non-motile cilia, known as primary cilia, extend from the surface of nearly all cell types; in NSPCs, they serve as sensory antennae facilitating several signaling cascades that enable NSPC response to regulatory cues in the neurogenic niches of specific periventricular regions (Kriegstein and Alvarez-Buylla, 2009). The genetic ablation of primary cilia in developing NSPCs results in hydrocephalus together with altered NSPC proliferation and decreased neurogenesis, which is unlikely to be explained in full by altered CSF circulation (Tong et al., 2014; Foerster et al., 2017).

The involvement of NSPCs in the pathogenesis of congenital hydrocephalus highlights the functional relationship between cerebral neurogenesis and the development of the brain CSF spaces. The genetic mutations, infections, and hemorrhage leading to congenital hydrocephalus have often been identified as pathological processes that can disrupt NSPC development. Instead of lifelong neurosurgical intervention, the precise targeting of NSPC defects by tailored pharmacological approaches is a promising strategy to prevent congenital hydrocephalus. A recent study has identified a possible hydrocephalus therapeutic target in the proteoglycan TSK, an ependymal-derived signal required for the proper levels of NSPC proliferation and survival in the mouse postnatal V-SVZ (Istiaq and Ohta, 2022). Strikingly, TSK-deficient mice display a patent LV enlargement along with several neurological deficits similar to those found in hydrocephalus patients (Ito et al., 2021). TSK directly binds to Wnt receptors *in vitro* and the LV expansion of TSK mutant mice could be rescued by injections of Wnt pathway inhibitors in the postnatal LV, suggesting that TSK modulates Wnt signaling in the V-SVZ, in agreement with the proposed role of this pathway in the regulation of NSPC proliferation (Kalani et al., 2008). Remarkably, point mutations in the TSK gene were found in hydrocephalus patients, which abolish both TSK binding to Wnt receptors and its function in the context of LV development, since a TSK variant carrying hydrocephalus-related mutations, unlike wild-type TSK, was not able to prevent hydrocephalus when expressed in ependymal cells or injected in the LV of TSK-deficient mice (Ito et al., 2021).

Another potential therapeutic target for hydrocephalus and other pathological conditions is LRP2, an endocytic receptor expressed by ependymal cells in the V-SVZ niche, where it is required to limit BMP pathway activation in NSPCs and support their proliferation and neurogenesis (Gajera et al., 2010). In humans, mutations in the *LRP2* gene cause Donnai-Barrow syndrome, a rare autosomal recessive disease associated with an heterogenous range of clinical symptoms including brain and ocular abnormalities (Kantarci et al., 2007). *LRP2* deletions were also associated with mild forms of holoprosencephaly (Rosenfeld et al., 2010), which were mirrored by the presence of an enlarged LV in the brain of LRP2-deficient mice, although the ventricular system and the ependymal layer did not show obvious alterations (Gajera et al., 2010). To gain insight into the cellular and molecular mechanisms underlying the neurogenic phenotype of LRP2-deficient mice, a recent study has compared the transcriptomic profiles of the neurogenic lineage in the wild type and in the mutant V-SVZ by means of single-cell RNA sequencing analysis of dissociated V-SVZ tissue (Zywitza et al., 2018). This analysis revealed a reduction of the NSC population, but not of TAPs and NBs, in the LRP2-deficient V-SVZ; transcriptomic profiling of cell cycle genes in these cell types suggested a decrease of TAP proliferation in LRP2 mutant mice, which was confirmed by BrdU incorporation assays. Differential gene expression analysis in specific neurogenic populations detected a downregulation of Wnt pathway genes in LRP2-deficient TAPs; reduced Wnt signaling in the mutant V-SVZ was confirmed by means of a Wnt-responsive reporter transgene (Zywitza et al., 2018). Taken together, these studies suggest that abnormal levels of Wnt signaling in NSPCs due to alterations in ependymal-derived signals may affect the neurogenic output of the V-SVZ niche as well as the morphogenesis of the LV; as a result, they highlight candidate extracellular determinants of hydrocephalus in the niche microenvironment that are potentially amenable to pharmacological treatments.

## Conclusion and Future Perspectives

The studies discussed in this review highlight the pivotal role of the extracellular cues produced by different cell types of the V-SVZ niche in the regulation of the proliferative state of NSPCs, which is crucial to modulate the neurogenic output of the niche and allow the lifelong production of new neurons. The existence of an important non-cell autonomous level of NPSC regulation is hardly surprising, given the influence, positive or negative, that various external stimuli can exert on the extent of V-SVZ neurogenesis. Yet, the complexity of this extrinsic regulation, which may be needed to give it sufficient robustness and sensitivity, is truly astonishing. As we have reviewed here, ependymal, vascular and LVCP cells are the sources of a variety of soluble, membrane-tethered or ECM-associated molecules acting at different levels of the neurogenic lineage, often with antagonistic activities; the contribution of signals provided by other V-SVZ cell types, such glial cells, immune cells and NSPCs themselves, and by the bloodstream, although not a focus of this review, is extensive and should also be taken into account. Untangling this complexity is clearly a daunting task, which will require major efforts for years to come. This task will be aided by the continuous improvement in several techniques, such as: conditional gene editing approaches, to manipulate these signals in specific cell types and time windows; single-cell transcriptomic, epigenomic and proteomic analyses, to define the genome-wide effects of these manipulations in specific cell populations; cell culture methods, to model specific components of the neurogenic niche *in vitro*; live imaging, to track individual NSPC behavior in time and space in different experimental conditions. Furthermore, key aspects that remain largely unaddressed include: the identification of the transcriptional regulators that sense and integrate the extracellular signaling pathways acting on NSPCs; the elucidation of the connections between the extracellular cues modulating NSPC activity and the cell cycle machinery; the characterization of the molecular changes in the extracellular niche milieu that are induced by different external stimuli affecting the neurogenic process, such as physical exercise, environmental enrichment, diet and tissue damage. Thanks to its well characterized structure and neurogenic lineage, its large neurogenic output, its functional relevance and its sensitivity to various stimuli, we expect that the rodent V-SVZ will continue to be a valuable model to investigate the extracellular regulation of postnatal neurogenesis. In turn, the knowledge obtained with this experimental paradigm could help to understand the apparent lack of neurogenic activity of the human V-SVZ beyond infancy (Sanai et al., 2011), and whether this niche may represent a target for translational therapies.
